# Thalamic Lesions: A Radiological Review

**DOI:** 10.1155/2014/154631

**Published:** 2014-07-02

**Authors:** Dimitri Renard, Giovanni Castelnovo, Chantal Campello, Stephane Bouly, Anne Le Floch, Eric Thouvenot, Anne Waconge, Guillaume Taieb

**Affiliations:** Service de Neurologie, CHU Nîmes, Hôpital Caremeau, Place du Pr Debré, 30029 Nîmes Cedex 4, France

## Abstract

*Background*. Thalamic lesions are seen in a multitude of disorders including vascular diseases, metabolic disorders, inflammatory diseases, trauma, tumours, and infections. In some diseases, thalamic involvement is typical and sometimes isolated, while in other diseases thalamic lesions are observed only occasionally (often in the presence of other typical extrathalamic lesions). *Summary*. In this review, we will mainly discuss the MRI characteristics of thalamic lesions. Identification of the origin of the thalamic lesion depends on the exact localisation inside the thalamus, the presence of extrathalamic lesions, the signal changes on different MRI sequences, the evolution of the radiological abnormalities over time, the history and clinical state of the patient, and other radiological and nonradiological examinations.

## 1. Introduction

The thalamus plays an important role in different brain functions including memory, emotions, sleep-wake cycle, executive functions, mediating general cortical alerting responses, processing of sensory (including taste, somatosensory, visual, and auditive) information and relaying it to the cortex, and sensorimotor control.

In this review, we will discuss the radiological characteristics of the diseases associated with preferential (and sometimes isolated), frequent, and less frequent thalamic involvement.

## 2. Vascular Lesions

### 2.1. Infarction

**Figure 1:**
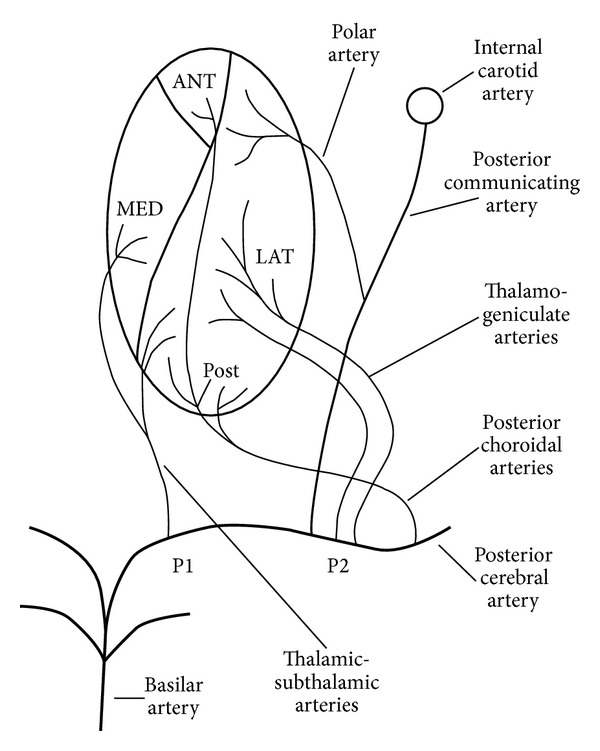
Schematic representation of the blood supply to the thalamus.

**Figure 2:**
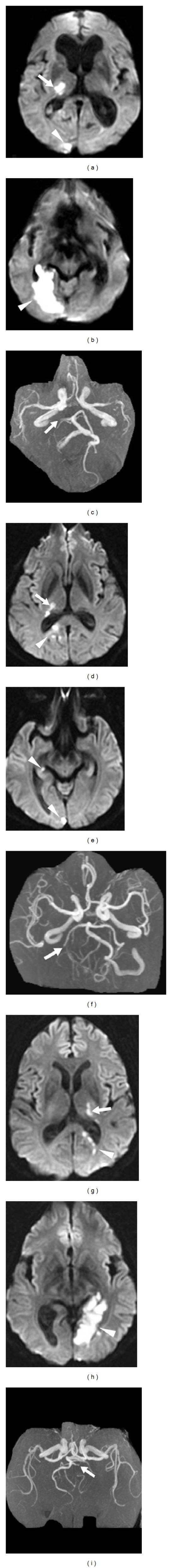
Thalamic infarctions in the thalamogeniculate artery territory (arrows in a, d, and g) in 3 different patients (patient 1 in a, b, and c; patient 2 in d, e, and f; patient 3 in g, h, and i) associated with other infarction areas in the ipsilateral posterior cerebral artery territories (arrowheads on a, b, d, e, g, and h) seen on DWI imaging due to posterior cerebral artery occlusion seen on TOF sequences (arrows on c, f, and i).

**Figure 3:**
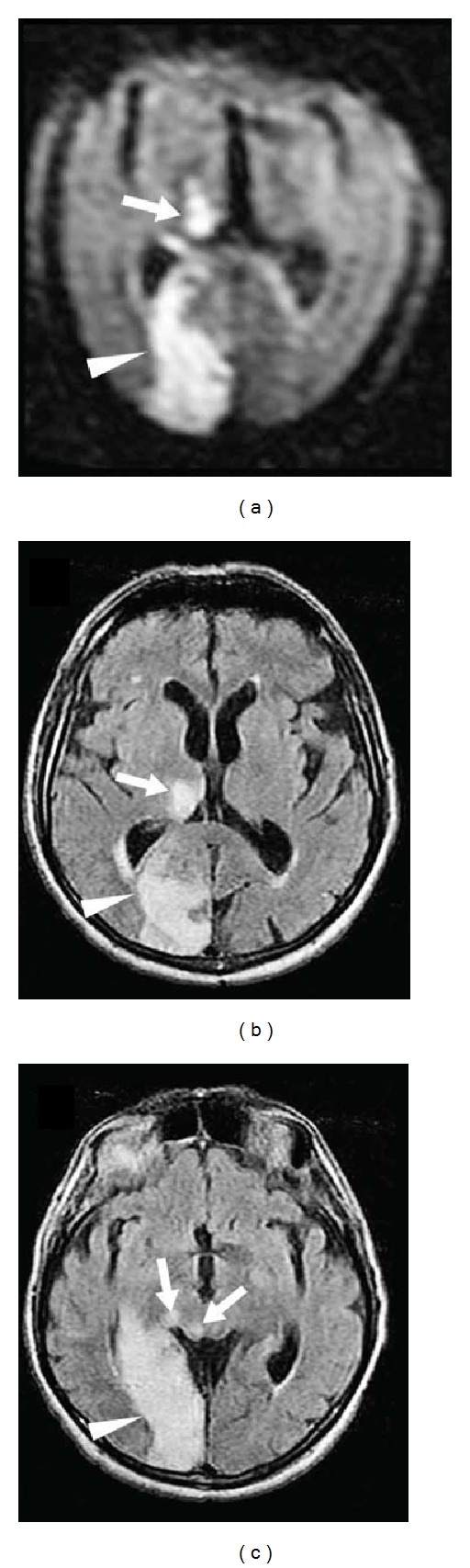
DWI (a) and FLAIR (b and c) imaging showing infarctions involving the territories of the right-sided posterior choroidal artery (a and b, arrows), the posterior cerebral artery (a, b, and c, arrowheads), the collicular artery, and the posteromedial choroidal artery (c, arrows).

**Figure 4:**
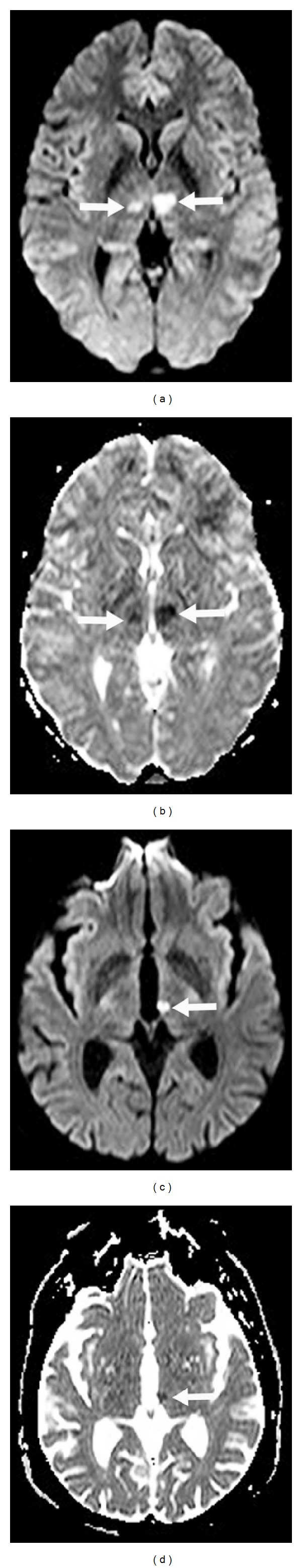
DWI (a and c) and ADC map (b and d) of 2 different patients (patient 1, a and b; patient 2, c and d) showing paramedian anterior thalamic infarction, bilateral in patient 1 and unilateral in patient 2.

The thalamus is mainly vascularised by the thalamogeniculate arteries (arising from the P2 portion of the posterior cerebral artery [PCA], supplying the ventrolateral region of the thalamus), the tuberothalamic artery (also called the polar artery, arising from the posterior communicating artery and supplying the anteromedial and the anterolateral region of the thalamus), the thalamosubthalamic arteries (also called the paramedian thalamic arteries, arising from the P1 portion of the PCA and supplying the medial region of the thalamus), and the posterior choroidal arteries (arising from the P2 portion of the PCA, supplying the posterior region of the thalamus including the pulvinar) [1] (Figure 1). Examples of thalamic infarctions in different arterial territories are shown in Figures 2, 3, and 4. In about one-third, the tuberothalamic artery is missing and its territory is supplied by the thalamosubthalamic arteries from the same side. The artery of Percheron is an anatomic variant where a single unpaired artery arising from the P1 portion of the PCA supplies the bilateral paramedian thalami and sometimes the rostral midbrain. Bilateral paramedian thalamic infarction (with or without rostral midbrain involvement) is observed when this artery is occluded. Multiple other anatomical variants of arterial supply of the thalamus have been described.

Considering that all arteries supplying the thalamus are terminal arteries, the thalamic infarctions have most often a lacunar aspect. The presence of other infarction areas in the PCA territory (supplying the occipital and the mesial temporal lobe) or their branches (e.g., collicular artery, posteromedial choroidal artery, penetrating midbrain arteries, etc.) could help to consider other mechanisms of stroke than lipohyalinosis or microatheroma.

Signal intensities and radiological evolution are like those seen in classical brain infarction.

### 2.2. Hemorrhage

**Figure 5:**
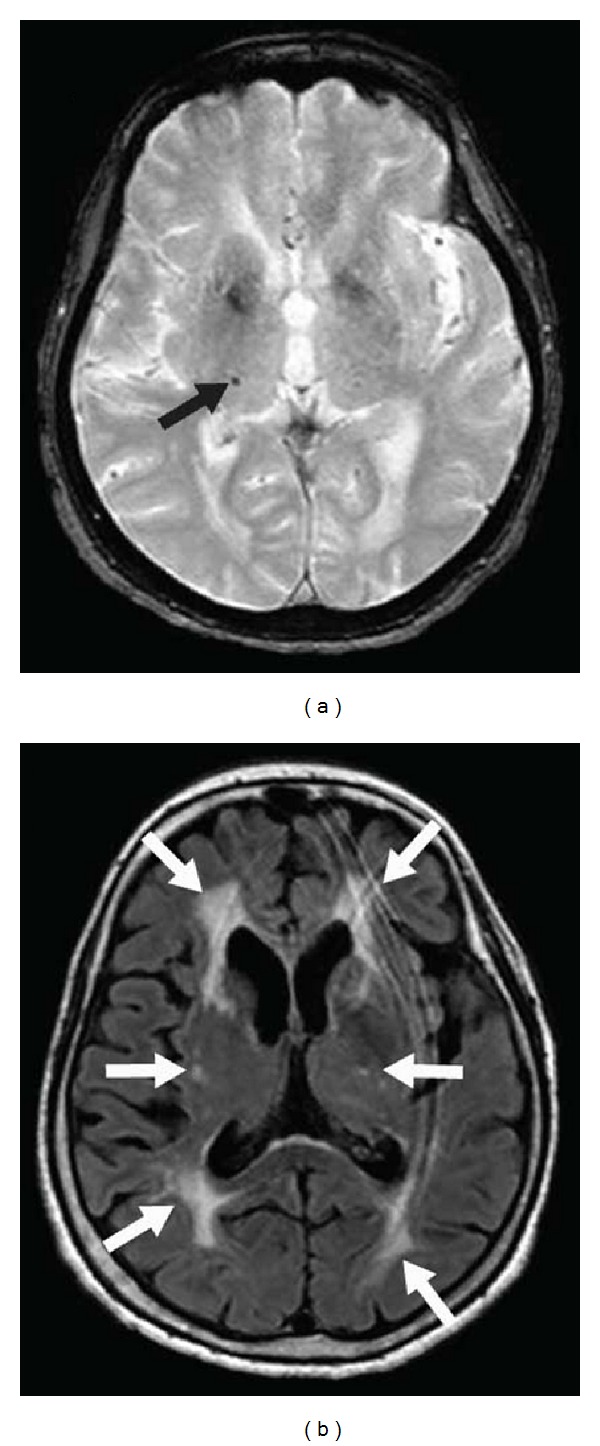
Gradient-echo (a) and FLAIR (b) sequences showing a right-sided thalamic microbleed (a) associated with diffuse ischemic white matter hyperintensities on FLAIR imaging (b) in a patient with severe and chronic arterial hypertension.

Thalamic microbleeds can be seen in small vessel disease (most often related to hypertension and associated with ischemic and hemorrhagic small vessel lesions in other brain areas) (Figure 5), after trauma (frequently also involving the corpus callosum, the midbrain, and/or the lobar white matter), secondary to initial infarction, or related to brain tumour. Amyloid-B deposition in cerebral amyloid angiopathy typically spares the deep perforating arteries making basal ganglia and thalamic microbleeds uncommon [2].

**Figure 6:**
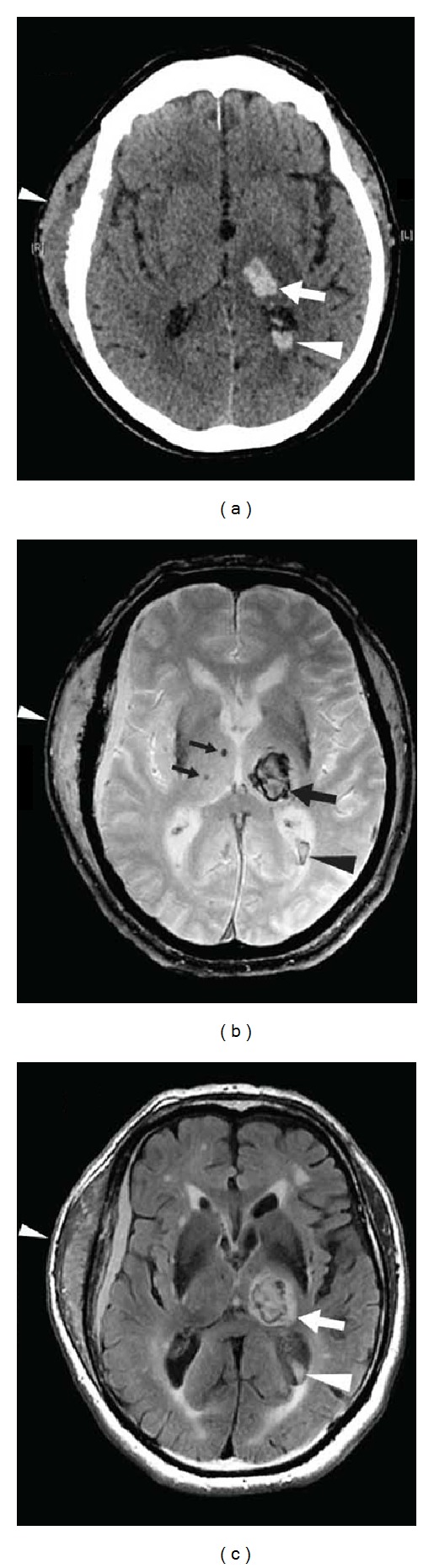
CT scan (a) and gradient-echo-weighted (b) and FLAIR (c) imaging showing a hypertension-related left-sided thalamic hemorrhage (large arrows on a, b, and c) and surrounding edema, complicated with a intraventricular hemorrhage (large arrowheads on a, b, and c) and associated with chronic hypertension-related microbleeds in the right thalamus (small arrows on b) and a subcutaneous and subdural hemorrhage (small arrowheads on a, b, and c) related to trauma (caused by acute right hemiplegie due to the thalamic hemorrhage).

Large thalamic (although less frequent than basal ganglia) hemorrhages are typically associated with hypertension (Figure 6). Because of the proximity of the third and the lateral ventricle, these lesions are often associated with intraventricular hemorrhage.

Thalamic vascular (e.g., cavernous) malformations can lead to both small- and large-size hemorrhages.

In both the acute and chronic phases of bleeding, prominent susceptibility effect, seen as hypointense “blooming” on T2* sequences, is typically seen. Susceptibility-weighted imaging (SWI) is even more sensitive than T2*-weighted imaging in detecting cavernous malformations (especially in multifocal/familial cases) and microbleeds [3]. Cavernous malformations most typically are not enhanced on gadolinium-injected T1-imaging, although slight enhancement may be observed. Cavernous malformations often show calcification, seen as hyperdensity on CT scan, and are hypointense on both T1- and T2-weighted sequences and profoundly hypointense on T2* and SWI imaging.

### 2.3. Venous Infarction

**Figure 7:**
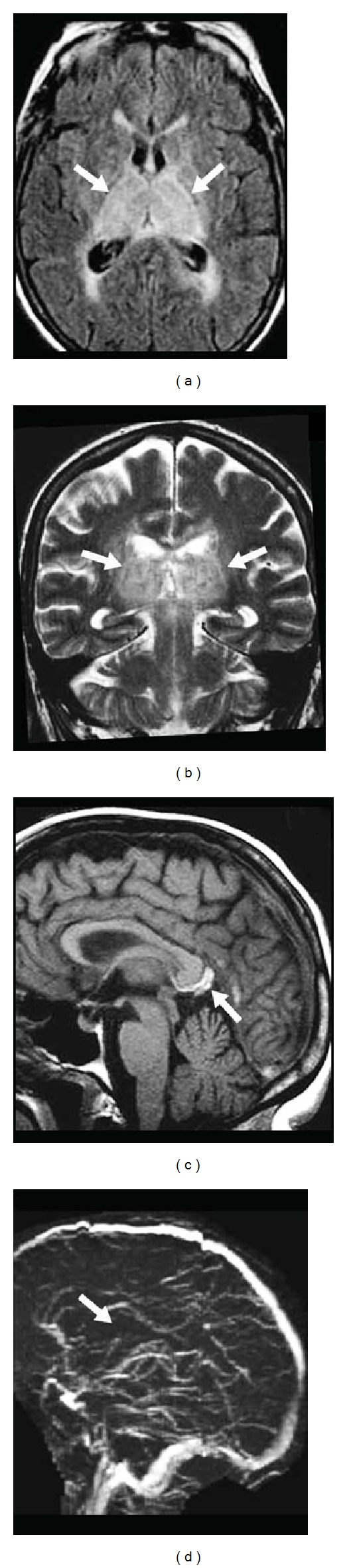
Bilateral thalamic vasogenic edema seen as hyperintensity on both axial FLAIR (a) and coronal T2-weighted (b) imaging due to venous thrombosis of the deep cerebral venous system. The venous thrombosis of the vein of Galen is seen as hyperintensity on sagittal unenhanced T1-weighted imaging (c), and lack of flow in the deep cerebral venous system is seen on MR venography (d).

Venous thrombosis of the deep venous system, the vein of Galen, or the straight sinus can lead to bilateral thalamic vasogenic edema (hyperintense on both DWI and ADC map) (Figure 7). These lesions may be complicated by cytotoxic edema (lowering or pseudonormalizing ADC values) and/or hemorrhage. The MRI signal intensity of the venous thrombus itself varies according to the time of imaging from the onset of thrombus formation. CT venography or TOF or gadolinium-enhanced MR venography is the most frequently used technique to show venous thrombosis.

## 3. Calcification

**Figure 8:**
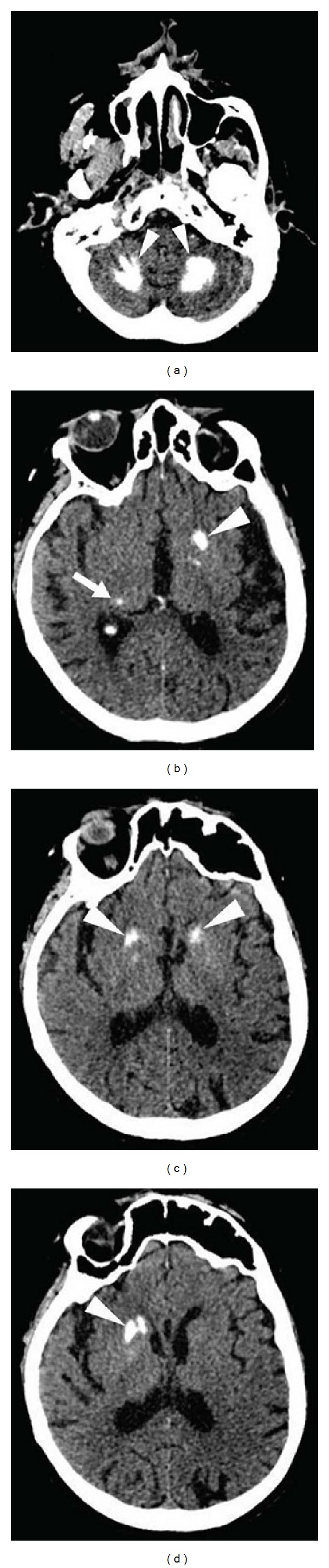
A patient with vitamin D deficiency with extensive cerebellar (a) and basal ganglia (arrowheads on b, c, and d) and thalamic (arrow on b) calcifications on CT.

**Figure 9:**
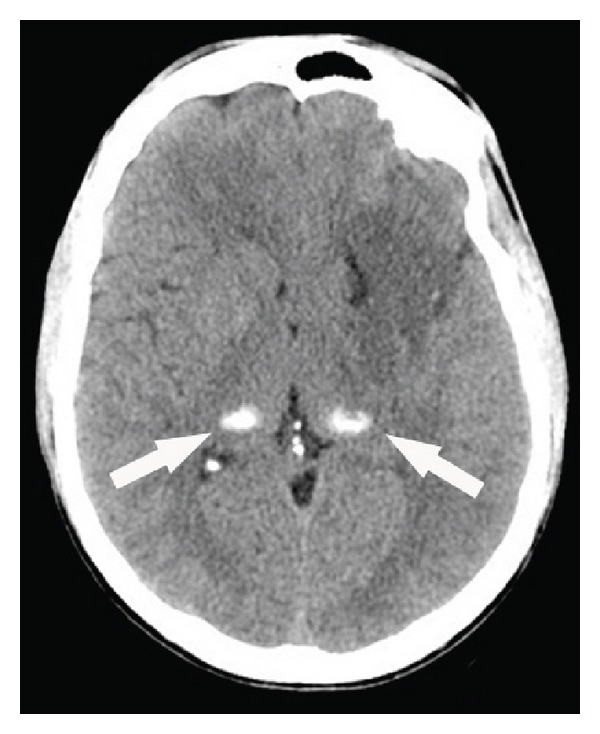
A patient with atypical Werner syndrome (i.e., a progeroid syndrome with Werner syndrome phenotype but without typical* RECQL2* mutation) due to a* LMNA* mutation showing bilateral thalamic calcifications on CT scan.

In a general population, incidence of basal ganglia and to a lesser degree thalamus calcifications increases with increasing age. Other causes of basal ganglia and thalamus calcifications include toxic (e.g., carbon monoxide, lead poisoning), postradiation/chemotherapy, infectious (e.g., tuberculosis, HIV, cytomegalovirus, toxoplasmosis, cysticercosis, and hydatidosis), metabolic (e.g., dysfunction in calcium metabolism), inherited (e.g., mitochondrial diseases, progeroid syndromes, Coat's plus syndrome, and leukoencephalopathy with calcifications and cysts), neonatal hypoxia, idiopathic (e.g., Fahr's disease) disorders, and vascular malformations (Figures 8 and 9).

## 4. Metabolic Diseases

### 4.1. Fabry Disease

**Figure 10:**
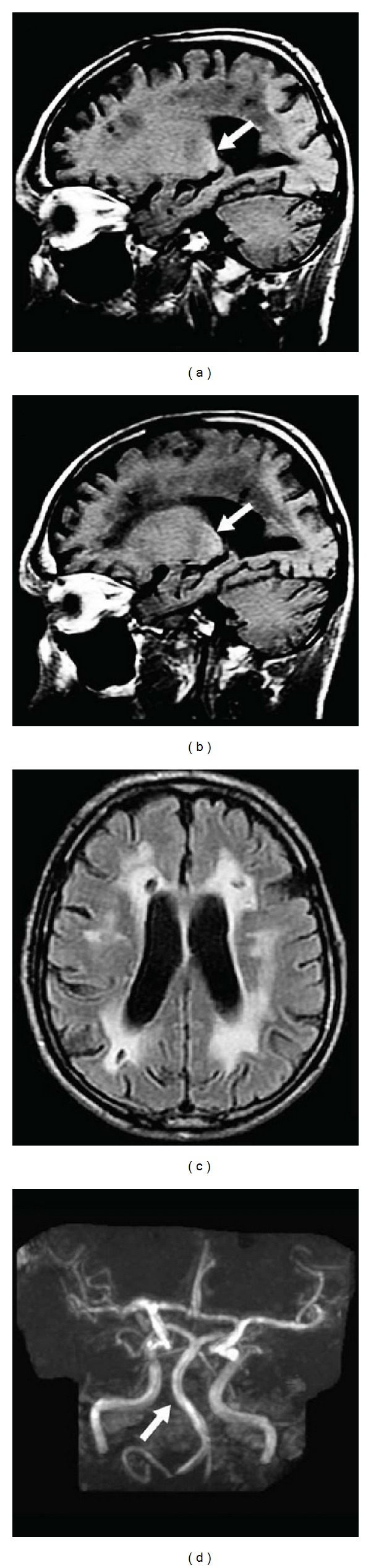
Sagittal unenhanced T1-weighted imaging (a and b) in a Fabry patient showing hyperintensity in the pulvinar, associated with ischemic leukoencephalopathy on FLAIR sequences (c) and vertebrobasilar dolichoectasia on TOF imaging (d).

Fabry disease is an X-linked lysosomal storage disorder due to alpha-galactosidase A gene mutation. The pulvinar sign, that is, an increased signal on unenhanced T1-weighted imaging involving the pulvinar, has been described in a portion (of especially male) Fabry patients (Figure 10) [4]. This T1 hyperintensity is thought to be due to the presence of calcification (or other mineralizing abnormalities). White matter lesions, brain infarctions (resulting probably from cardiac embolism, large and small vessel disease), lobar hemorrhages (attributed to hypertension), and microbleeds (attributed to hypertension and/or small vessel disease) have been reported in both male and female Fabry patients [5].

### 4.2. Osmotic Demyelinating Syndrome

The osmotic demyelinating syndrome, formerly called central pontine myelinolysis (because of the frequent pontine involvement) or extrapontine myelinolysis (when other than pontine lesions are present), can be seen with any kind of osmotic gradient changes. The thalamus is one of the frequent sites (together with the cerebellum, basal ganglia, cerebral white matter, hippocampus, and the corpus callosum) of extrapontine localisation. The lesions are T2/FLAIR hyperintense and T1 hypointense in the acute phase, often resolved after the acute phase. Hemorrhage and contrast enhancement are rare. Lesions sometimes occur with a certain delay after the onset of clinical symptoms. Lesions are rarely observed in absence of clinical abnormalities. Increased DWI signal and heterogeneous signal changes on ADC map often accompany the changes on T1- and T2-weighted imaging [6].

### 4.3. Wernicke Encephalopathy

**Figure 11:**
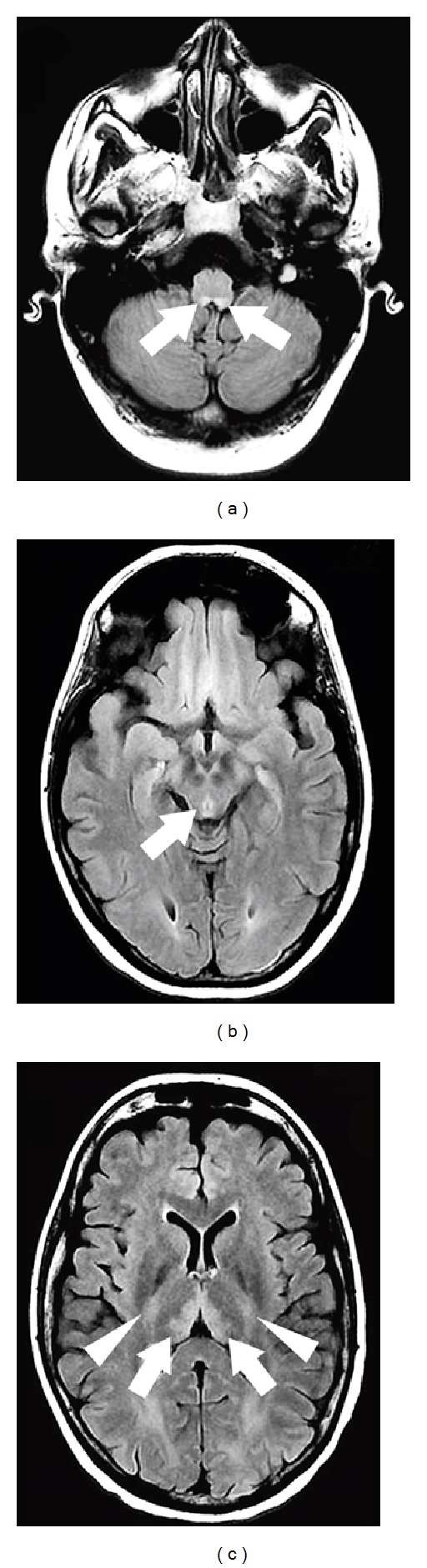
A patient with Wernicke syndrome showing FLAIR hyperintensities in the hypoglossal nuclei (a), the periaqueductal gray matter and mesencephalic tectum (b), the medial part of the thalami (arrows, c), and the pyramidal tracts (arrowheads, c).

**Figure 12:**
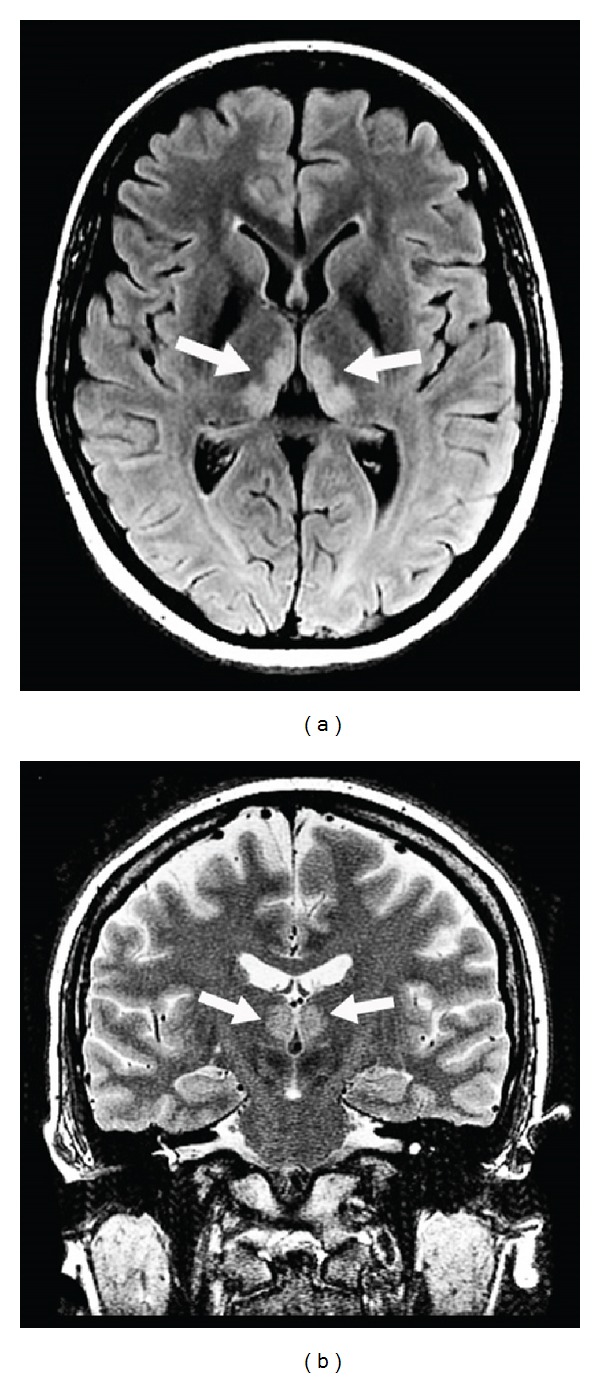
A patient with Wernicke syndrome showing hyperintensities in the medial part of the thalami on axial FLAIR (a) and coronal T2-weighted (b) imaging.

In Wernicke encephalopathy, frequently involved brain areas include the thalamus, the periaqueductal gray matter, the mamillary bodies, the hypothalamus, and the perirolandic regions. Involvement of the cranial nerve nuclei, the frontal and the parietal lobes, and the corpus callosum is less frequent. The medial part is the most typically involved portion of the thalamus (Figures 11 and 12). Lesions are most often symmetrical and best seen as hyperintensity on T2/FLAIR sequences. Enhancement (especially in alcoholic patients) and/or reduced diffusion in the acute phase can been sometimes observed. Hemorrhagic lesions have been reported in catastrophic cases.

### 4.4. Inherited Metabolic Disorders

Thalamic MRI signal changes can be seen in several inherited metabolic disorders including Wilson disease, Leigh syndrome, Krabbe disease, maple syrup urine disease, Canavan disease, Alexander disease, and gangliosidosis. In these disorders, associated signal abnormalities in other brain areas (e.g., white matter, basal ganglia, and brainstem) are frequently observed. The MRI signal often changes over time in these diseases. Radiological abnormalities often show an increased T2 and a decreased T1 signal. The reverse (i.e., decreased signal on T2- and increased signal on T1-weighted imaging) can be seen in gangliosidosis. In early stage Krabbe disease, decreased signal is usually observed on both T1- and T2-weighted imaging, whereas increased signal is present in chronic stage disease on these sequences. Mixed T2 signal is typically seen in Wilson disease. Restricted diffusion may be observed in maple syrup urine, Canavan, and acute Leigh disease.

Gangliosidosis affects preferentially the thalami, seen as hyperdensity on unenhanced CT scan. On MRI, lesions are hyperintense on T1-weighted and hypointense on T2-weighted imaging, often associated with leukoencephalopathy and cerebellar atrophy.

In neurodegeneration with brain iron accumulation related-disorders, the presence of thalamic hypointensities on T2*-weighted or SWI imaging is suggestive of aceruloplasminemia and neuroferritinopathy [7].

## 5. Reversible Posterior Leukoencephalopathy Syndrome

Risk factors for reversible posterior leukoencephalopathy syndrome include immunosuppressive and cytotoxic agents, hypertension, eclampsia, and metabolic abnormalities.

**Figure 13:**
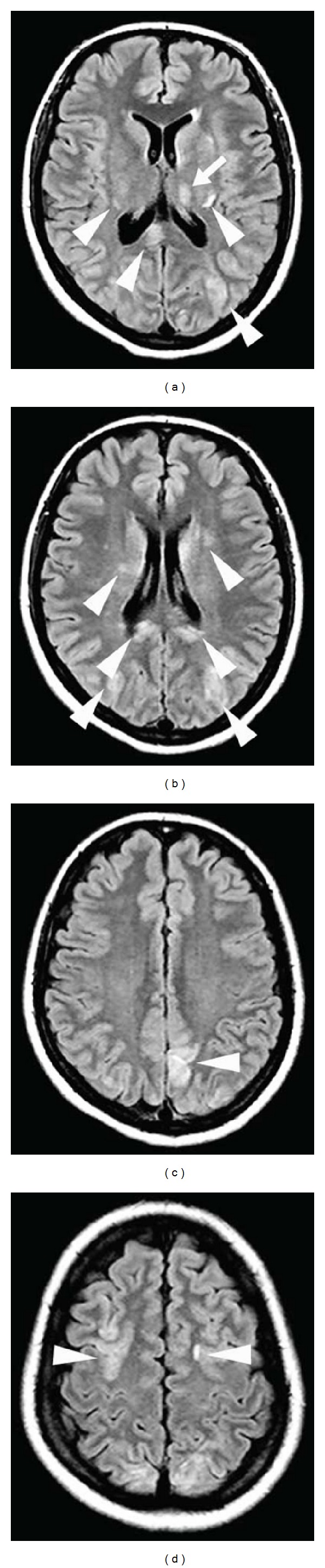
A patient with preeclampsia-related reversible posterior leukoencephalopathy syndrome with cortical and subcortical (arrowhead on a, b, c, and d) vasogenic edema seen as hyperintensity on FLAIR sequences also present in the left thalamus (arrow, a).

Brain imaging typically shows bilateral white matter lesions in the occipital and posterior parietal lobes. Watershed areas between middle and posterior cerebral arteries are frequently involved. However, associated involvement of grey matter and other brain regions including frontal and temporal lobes, brainstem, cerebellum, basal ganglia, thalamus, and corpus callosum are frequently seen (Figure 13).

MRI characteristics are indicative of vasogenic oedema (hyperintense on T2, FLAIR, and ADC sequences, iso- or slightly hyperintense on DWI, and iso- to hypointense on T1-weighted images). ADC values seem to be more sensitive to show brain abnormalities than findings on conventional T2 and FLAIR sequences.

Associated infarction (due to decreased cerebral blood flow in areas of massive oedema and elevated tissue perfusion pressure), hemorrhage (especially when associated with hypertension), and/or gadolinium-enhancement are sometimes observed. In case of infarction, the affected regions show highly increased signal on DWI and pseudonormalized or decreased signal on ADC map. In uncomplicated patients, regression (at least partially) of the radiological abnormalities is typically seen after discontinuation of the offending drug and the treatment of elevated blood pressure.

## 6. Demyelinating Lesions

### 6.1. Multiple Sclerosis

**Figure 14:**
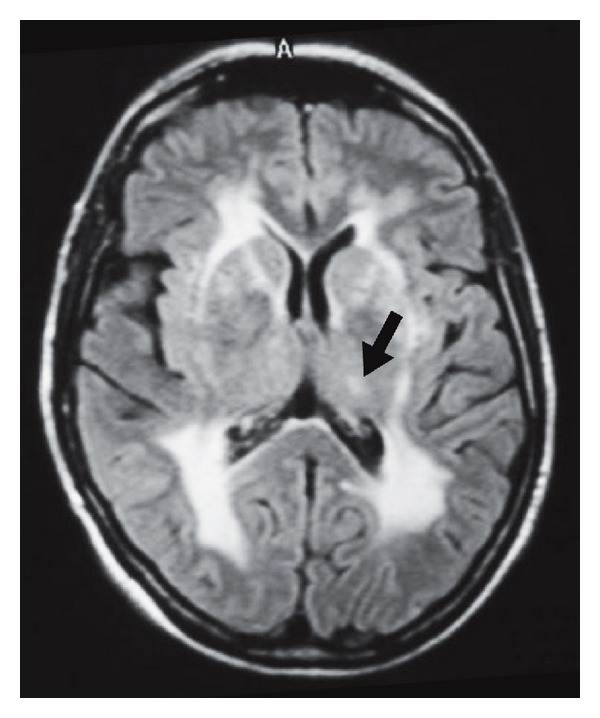
A patient with longstanding multiple sclerosis with diffuse leukoencephalopathy and a left thalamic demyelinating lesion (a) on FLAIR imaging.

Thalamic lesions are rare but have been reported especially in long-standing multiple sclerosis (Figure 14) [8]. Multiple sclerosis lesions appear typically as T2 and FLAIR hyperintensity.

On high-field strength MRI, diffuse decreased thalamic and putaminal T2 signal (also called “black T2”) can be observed in multiple sclerosis patients, likely to be caused by increased iron accumulation [9, 10].

### 6.2. Acute Disseminated Encephalomyelitis

Acute disseminated encephalomyelitis is a monophasic postinfectious or postvaccination disorder not requiring long-term treatment. Radiological features overlap partially with multiple sclerosis. However, corpus callosum and periventricular lesions are less frequent and thalamic and basal ganglia lesions far more frequent in acute disseminated encephalomyelitis than in multiple sclerosis. In acute disseminated encephalomyelitis, gadolinium-enhanced T1 imaging typically shows enhancement of all (or nearly all) lesions.

### 6.3. Neuromyelitis Optica Spectrum Disorders

Classically, neuromyelitis optica was thought to show no or only discrete brain MRI abnormalities. Recent studies, however, analysing systematically brain lesions in neuromyelitis optica showed that these lesions are more frequent with somewhat different radiological characteristics (i.e., more often diffuse, heterogeneous, and cystic and with blurred margins) than seen in multiple sclerosis. When present, the periventricular white matter is most frequently involved. Thalamus (and basal ganglia) involvement is rare but has been reported [11].

## 7. Nondemyelinating Inflammatory Diseases

**Figure 15:**
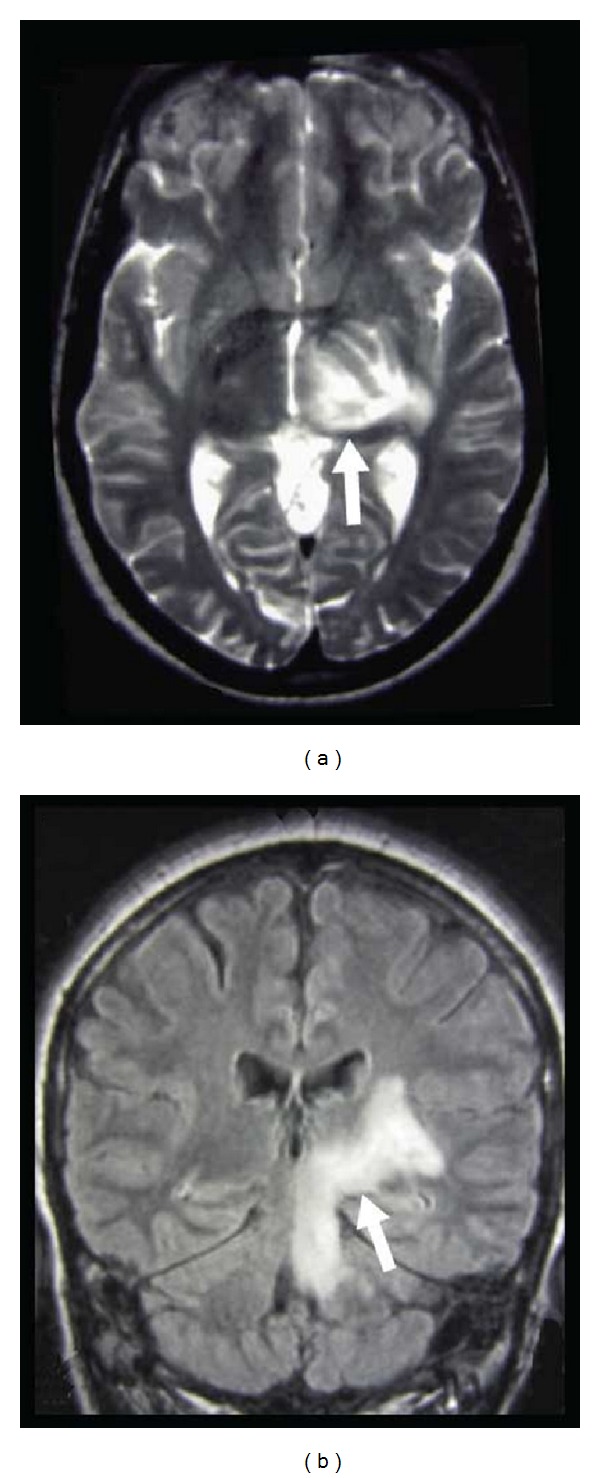
A patient with neuro-Behcet showing a left-sided hyperintense lesion on axial T2-weighted (a) and coronal FLAIR (b) imaging involving the cerebellum, the midbrain, and the inferior part of the thalamus (arrows), the internal capsule, and the putamen.

Nondemyelinating inflammatory diseases, such as venous vasculitis (e.g., Behcet disease) or connective tissue diseases (e.g., Sjögren's syndrome), sometimes involve the thalamus (Figure 15) [12, 13]. These lesions are most often hyperintense on T2 and FLAIR sequences and are sometimes enhanced on gadolinium-injected T1-weighted imaging. Thalamic involvement has been rarely observed in other autoimmune-related disorders such as Bickerstaff brainstem encephalitis or paraneoplastic encephalitis [14–16].

## 8. Trauma

### 8.1. Diffuse Axonal Injury

**Figure 16:**
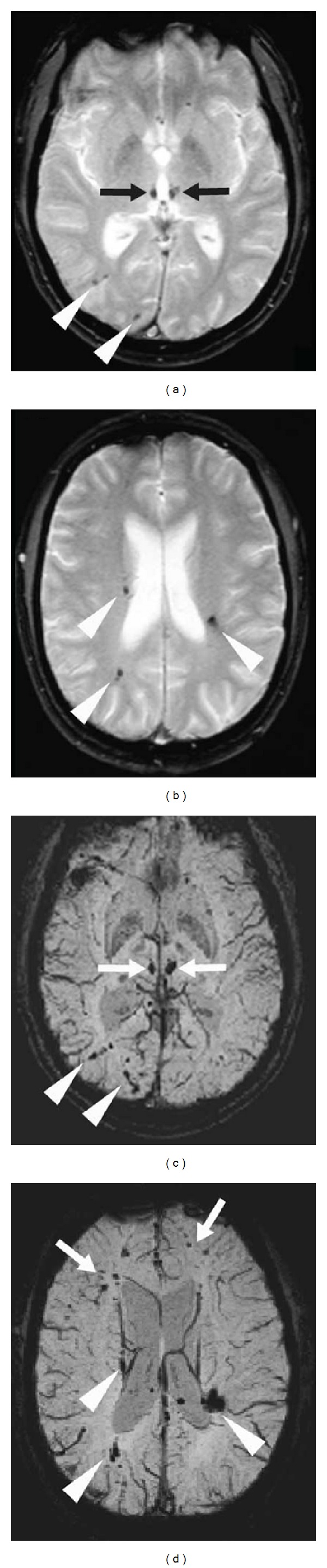
A patient after a major head trauma showing diffuse axonal injury with small hemorrhagic lesions in the medial part of both thalami (arrows, a) and multiple lesions near the cortico-subcortical junction (arrowheads, a and b) seen as hypointensity on gradient-echo-weighted imaging (a and b). Multiple additional lesions can be seen on SWI imaging (c and d) demonstrating the superiority of SWI imaging in diffuse axonal injury.

Diffuse axonal injury typically involves the corpus callosum, the midbrain, and the lobar white matter. Thalamus and basal ganglia involvement has been described less frequently (Figure 16). Lesions, often multiple, are best seen on DWI and FLAIR sequences as hyperintense signal, with frequently associated hemorrhagic hypointense lesions on T2*-weighted images (and even better seen on SWI sequences). On ADC map, lesions may be hypointense indicating cytotoxic edema. Often associated radiological manifestations of head trauma (including epidural, subdural, subarachnoid, or intraventricular hemorrhage, contusion) are present. Diffuse axonal injury lesions tend to reduce in number and volumes over time.

## 9. Neoplastic

### 9.1. Glioblastoma Multiforme

**Figure 17:**
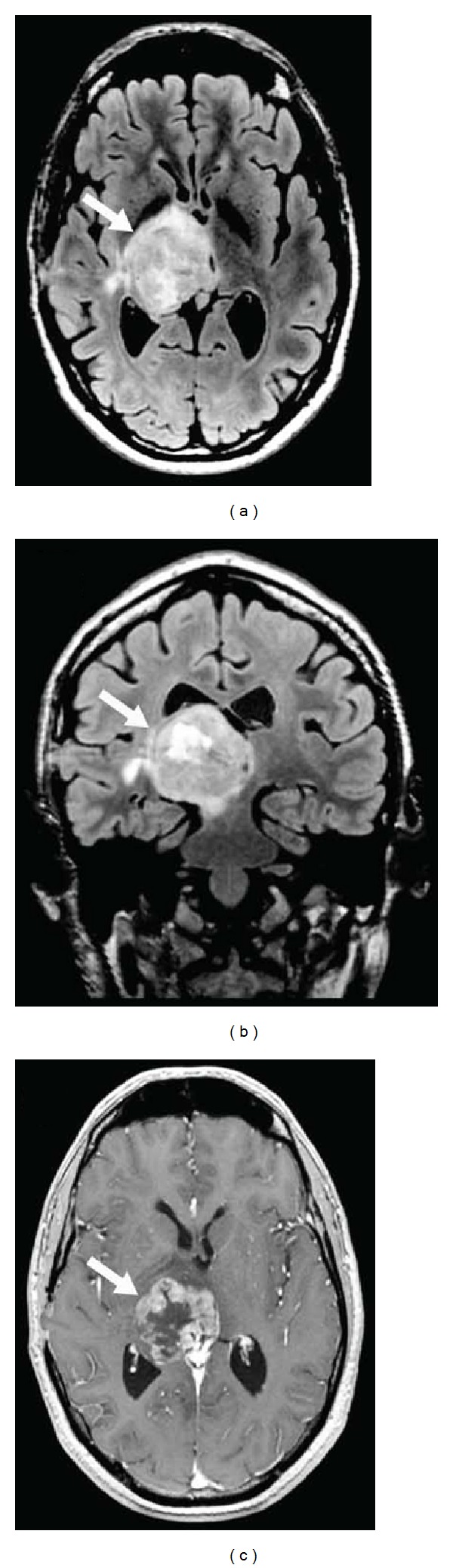
A patient with glioblastoma multiforme in the right thalamus seen as hyperintensity on axial (a) and coronal (b) FLAIR imaging, with gadolinium-enhancement on axial T1-weighted imaging (c).

Glioblastoma multiforme commonly affects the thalamus (Figure 17). The MRI signal is most typically heterogeneous, iso- to hypointense (especially when necrosis is present) on T1 sequences, and hyperintense on T2 and FLAIR imaging. Central necrosis, perilesional vasogenic (T2/FLAIR/ADC hyperintense) edema, and strong (solid, nodular, patchy, or “closed-ring”) enhancement are typical. Sometimes, hemorrhage occurs inside the tumour.

### 9.2. Gliomatosis Cerebri

**Figure 18:**
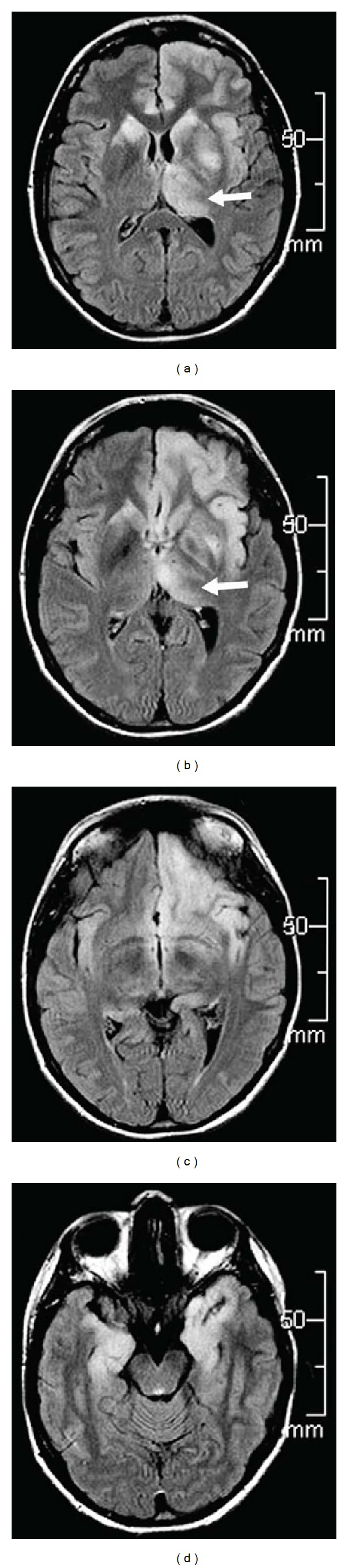
A patient with gliomatosis cerebri involving the left thalamus (arrows, a and b), the bilateral basal ganglia (a and b), the left frontal lobe (a, b, and c), and both temporal lobes (d), seen as hyperintensity on FLAIR sequences.

In gliomatosis cerebri, diffuse white matter infiltration (best seen as homogenous T2 and FLAIR hyperintensity, hypointense on T1-weighted imaging) involving two or more lobes is observed with enlargement of the involved structure. Absent (or minimal) enhancement on gadolinium-injected T1-weighted imaging is typical. Associated thalamic, basal ganglia, and/or corpus callosum involvement is frequently observed (Figure 18).

### 9.3. Lymphoma

**Figure 19:**
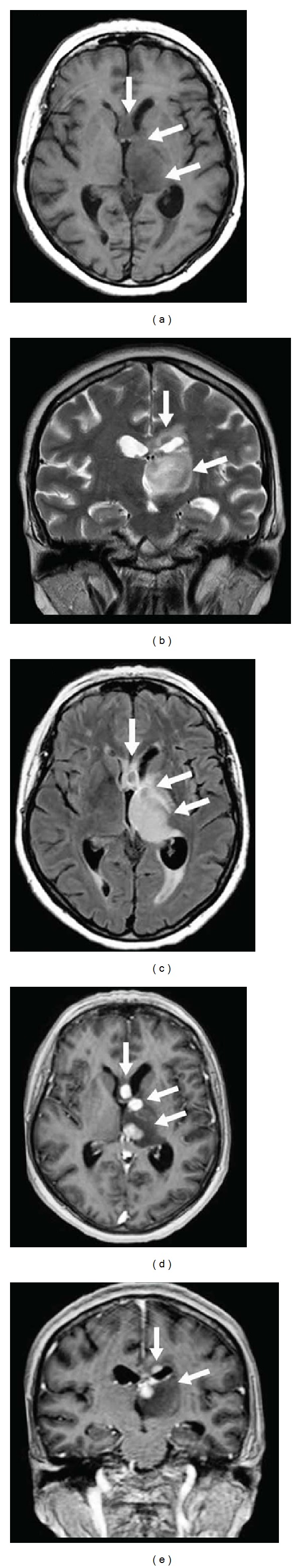
A patient with primary CNS lymphoma involving the left-sided thalamus, the internal capsule, and the anterior part of the corpus callosum seen as hypointensity on axial unenhanced T1-weighted imaging (a), hyperintensity on coronal T2-weighted (b) and axial FLAIR (c) imaging, with multifocal enhancement on axial (d) and coronal (e) gadolinium-enhanced T1-weighted imaging.

Lymphoma often involves the periventricular white matter, the thalamus, the basal ganglia, and the corpus callosum. Lymphomas are iso- or hypointense on unenhanced T1 sequences and hyperintense on T2/FLAIR imaging, with homogenous contrast enhancement in absence of central necrosis (Figure 19). In immunocompromised and rarely in nonimmunocompromised patients, contrast enhancement is rather peripheral than homogeneous or may be less evident or even absent. Surrounding edema as well as central necrosis may be seen in HIV-related lymphoma. In contrast to glioblastoma, there is less (or absent) peritumoral edema, and necrosis and hemorrhage are less common in lymphoma. Reduced diffusion has been reported occasionally. Lymphoma responds often dramatically (and frequently disappears on MRI) but temporarily to steroid treatment and radiation therapy.

### 9.4. Metastasis

Metastatic thalamic lesions are infrequent and are most often seen in the presence of other metastatic brain lesions. Lesion characteristics depend on the primary malignancy but are most often present with mass effect, surrounding edema, and contrast enhancement.

## 10. Infection

### 10.1. Encephalitis

Rare cases of infectious encephalitis involving the thalamus have been described. In these cases, thalamic lesions often coexist with more typical encephalitis lesions [17]. Lesions are most often hyperintense on T2 and FLAIR imaging. Associated diffusion restriction, hemorrhage, or gadolinium-enhancement can be sometimes observed.

**Figure 20:**
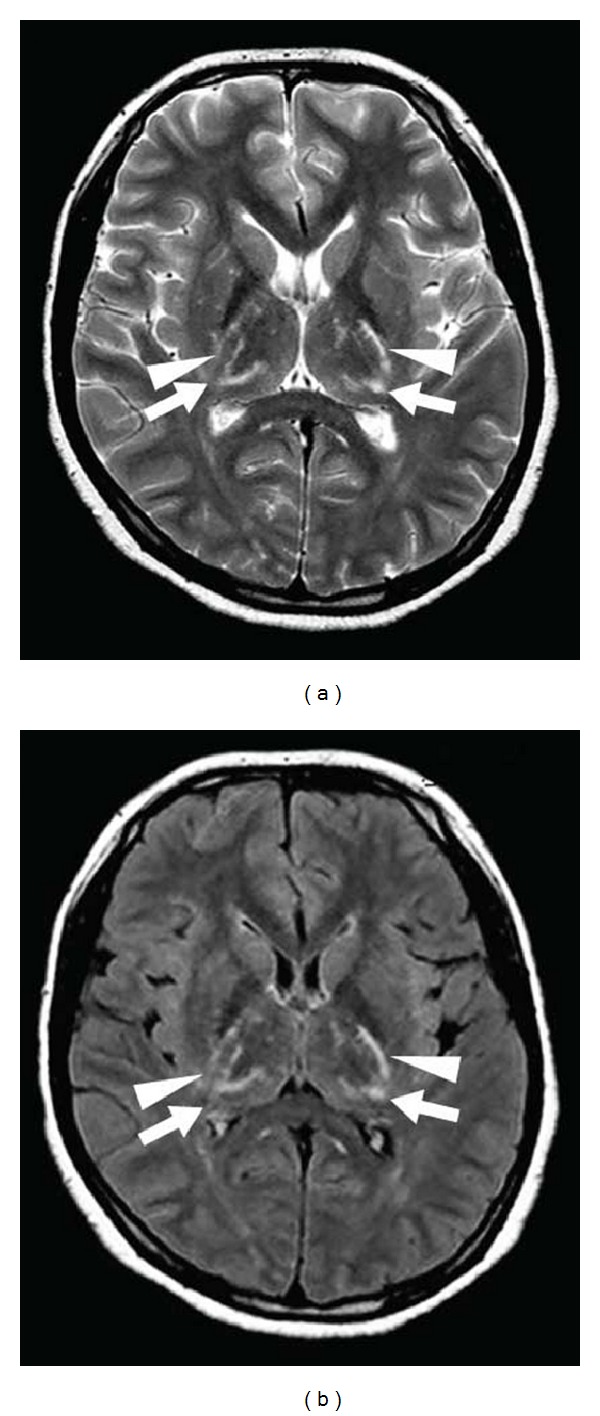
A patient with Mycoplasma pneumoniae-related acute necrotizing encephalitis involving symmetrically the posterior part of the internal capsule (arrowheads) and the posterolateral portion of the thalamus (arrows) on both sides, seen as hyperintensity on both T2-weighted (a) and FLAIR (b) imaging.

In postinfectious (e.g., influenza A, parainfluenza, and Mycoplasma pneumoniae) acute necrotizing encephalopathy, often occurring in children, the thalamus is preferentially involved (often with associated brainstem, basal ganglia, cerebellum, or periventricular white matter lesions), seen as hyperintensity on T2-weighted and FLAIR imaging, and sometimes complicated with hemorrhage (Figure 20). Familial or recurrent cases of infection-triggered acute necrotizing encephalopathy can be caused by a* RANBP2* gene mutation [18].

### 10.2. Brain Abscess

**Figure 21:**
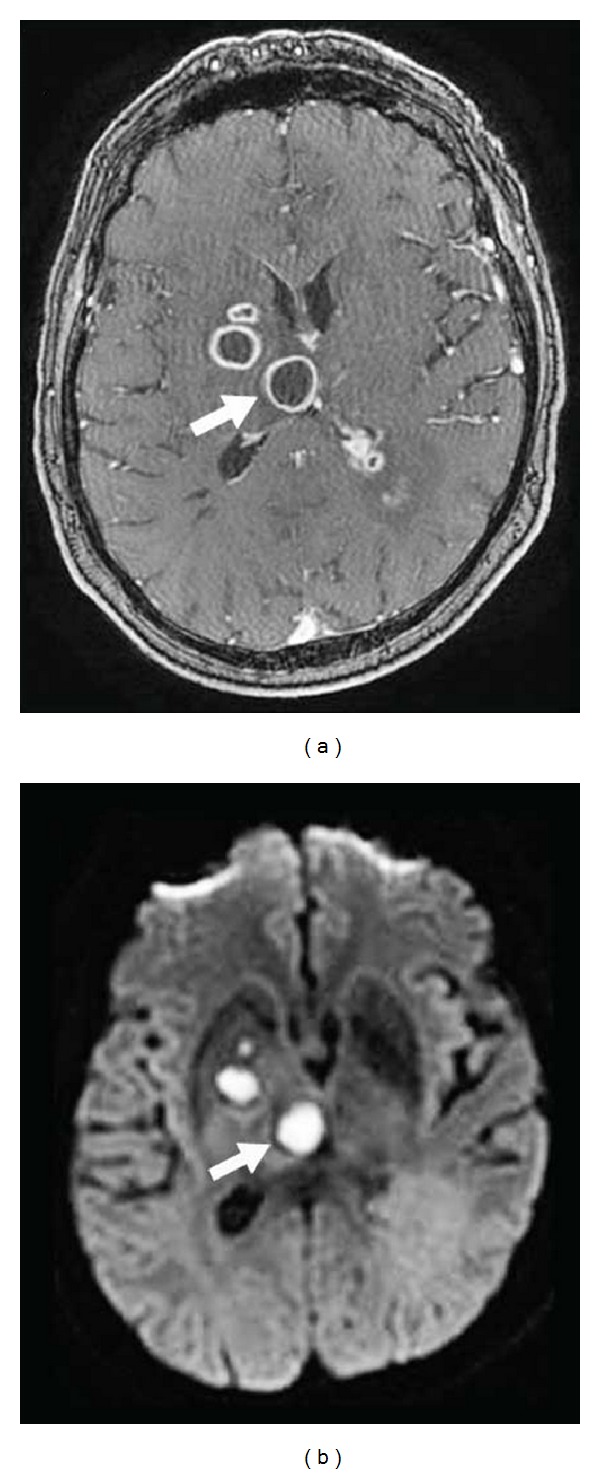
A patient with several brain abscesses involving the right thalamus (arrows) and the basal ganglia, with ring enhancement on gadolinium-enhanced T1-weighted imaging (a) and restricted diffusion seen as hyperintensity on DWI imaging (b).

Brain abscesses are usually located supratentorially at the gray-white matter junction with radiological characteristics varying with the stage of abscess development. Deep gray matter (including thalamic) involvement is sometimes observed (Figure 21). Typical MRI characteristics include restricted diffusion on diffusion-weighted imaging (because of a high protein content), ring enhancement on gadolinium-enhanced T1-weighted imaging, and surrounding (T2 and FLAIR hyperintense) edema.

### 10.3. Progressive Multifocal Leukoencephalopathy

**Figure 22:**
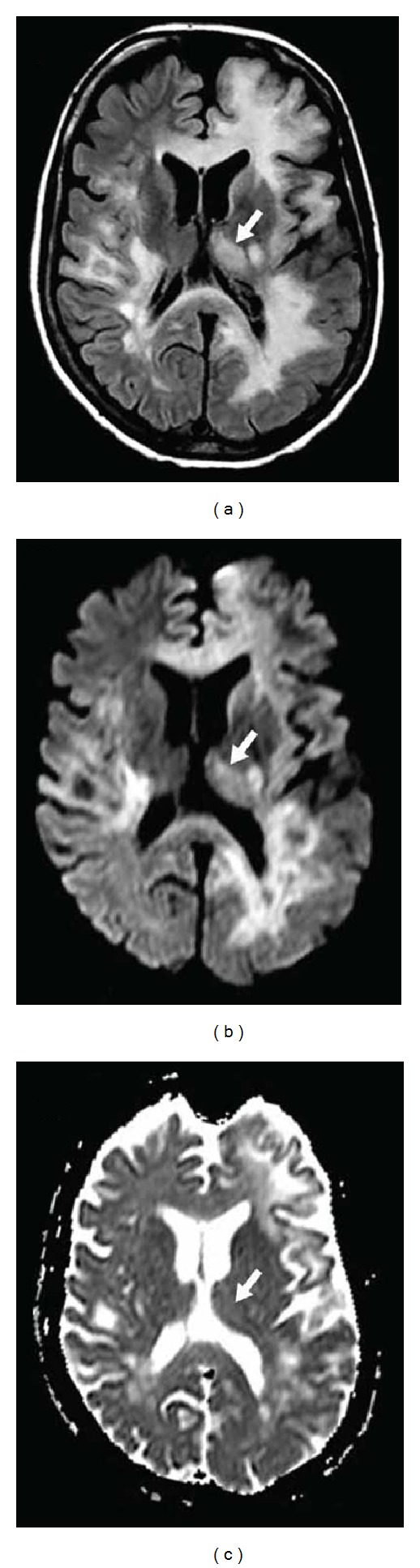
Diffuse white matter and associated left frontal cortical and left thalamic (arrows) involvement seen as hyperintensity on FLAIR (a), DWI (b), and ADC map (c) in a patient with natalizumab-related (for multiple sclerosis) progressive multifocal leukoencephalopathy.

The JC-virus-related progressive multifocal leukoencephalopathy typically occurs in immunocompromised patients and has a high mortality. These T2/FLAIR hyperintense and T1 hypointense lesions are uni- (especially in the early stage) or multifocal, usually without mass effect, and involve mainly the subcortical white matter although basal ganglia, thalamus and cortex involvement are sometimes encountered (Figure 22). Lesions are often hyperintense on DWI. Contrast enhancement is most often absent although faint enhancement can be sometimes observed at the periphery. Enhancement seems to be more frequent in natalizumab-induced progressive multifocal leukoencephalopathy cases. In these patients, small punctuate T2-hyperintense lesions in the immediate vicinity of the main lesions are often seen. T1-hyperintense signals can be found during and after the immune reconstitution inflammatory syndrome phase of progressive multifocal leukoencephalopathy. Surviving patients typically show profound atrophy of the involved brain structures in the chronic phase of the disease.

### 10.4. Creutzfeldt-Jakob Disease

**Figure 23:**
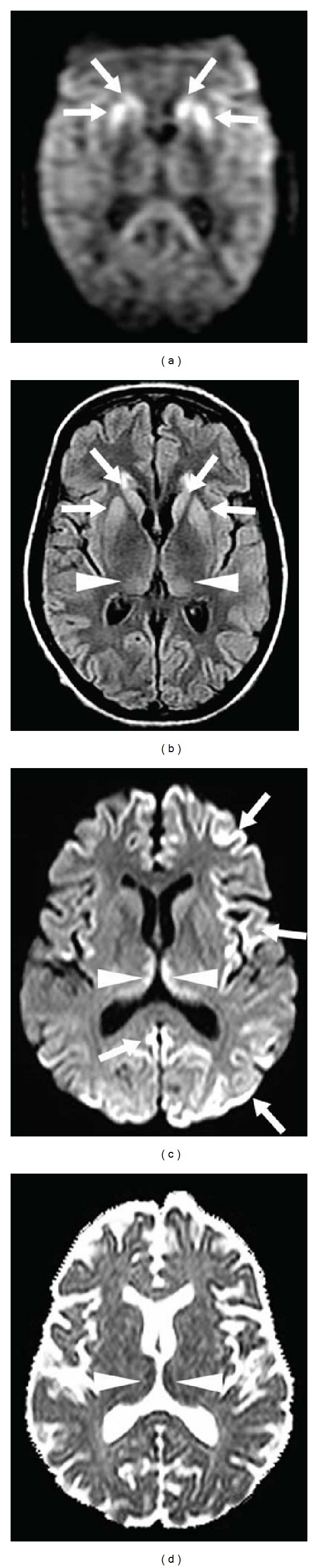
Two different sporadic CJD patients (patient 1, a and b; patient 2, c and d) showing in patient 1 bilateral caudate nucleus and putaminal (arrows) and to a lesser degree posteromesial and pulvinar thalamic (arrowheads) hyperintensities on DWI (a) and FLAIR imaging (b) and in patient 2 bilateral posteromesial thalamic (arrowheads) and multifocal cortical (arrows) hyperintensities on DWI (c), seen as hypointensity on ADC map (d). Anterior predominance of the thalamic involvement can be seen in patient 2.

Increased DWI and/or FLAIR signal in the basal ganglia, the thalamus and/or, the cerebral cortex is typical in Creutzfeldt-Jakob disease (Figure 23). MRI abnormalities, together with the presence of clinical signs, periodic sharp wave complexes on electroencephalogram, and 14-3-3 protein in the cerebrospinal fluid, make a premortem diagnosis of probable sporadic Creutzfeldt-Jakob disease possible [19]. MRI signal changes in early stage disease can be absent or very subtle. DWI seems to be more sensitive than FLAIR sequences to detect early signal changes [20].

In sporadic Creutzfeldt-Jakob disease, there is an anterior predominance of basal ganglia MRI changes (i.e., caudate nucleus is more frequently and more severely involved than the lentiform nucleus, whereas the thalamus is the least frequently and severely involved deep gray matter structure). Deep grey matter signal changes are most often bilateral (asymmetrical or symmetrical) although unilateral involvement can be seen. In case of thalamic involvement, signal abnormalities are most typically seen in the posteromesial portion. In young sporadic Creutzfeldt-Jakob patients, thalamic involvement is sometimes more severe than in the anterior basal ganglia structures. In these young patients, some authors report signal changes predominant in the anterior portion of the thalamus.

In the variant Creutzfeldt-Jakob disease, the thalamus is the most frequently involved deep grey matter structure with typical bilateral symmetrical involvement (the so-called “hockey-stick” sign) with the posterior portion of the thalamus (pulvinar) as the most hyperintense substructure (i.e., more hyperintense than the anterior part of the thalamus) on DWI/FLAIR imaging [21].

## 11. Laminar Necrosis

**Figure 24:**
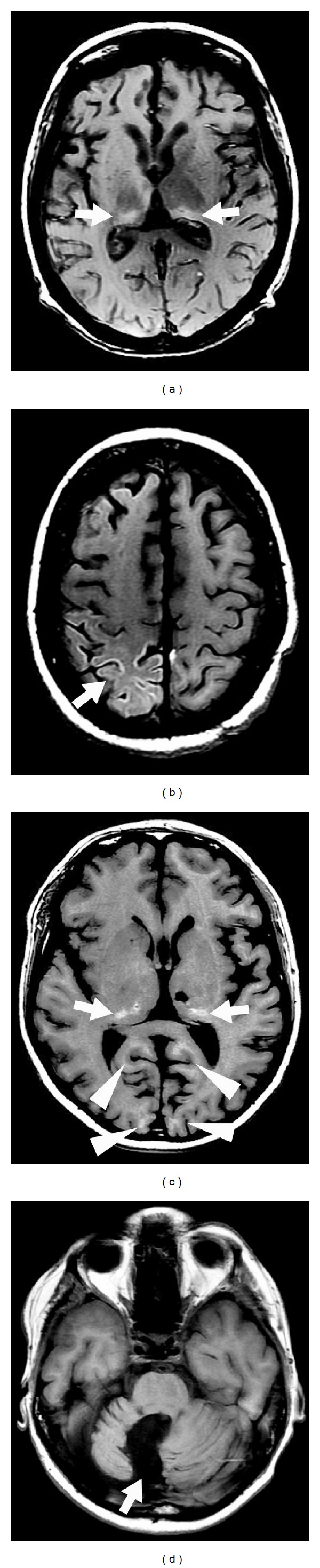
T1-weighted MRI of patient 1 (a and b) after generalized status epilepticus and patient 2 (c and d) with a history of cerebellar astrocytoma treated by surgical resection and large field radiation therapy including the occipital lobes and both thalami showing laminar necrosis of both thalami (a and c, arrows) associated with laminar necrosis in the right-sided parietal cortex (b, arrows) and in the bilateral occipital cortex (c, arrowheads). Panel (d) shows the cerebellar cavity due to cerebellar astrocytoma resection.

Laminar necrosis typically involves the cortex but has also been reported in the basal ganglia and the thalami (Figure 24) [22]. Laminar necrosis appears as hyperintensity on unenhanced T1-weighted imaging. The proposed mechanism is cytolysis, necrosis, edema, followed by resorption, and phagocytosis of necrotic material, resulting in fat-laden macrophages depositions explaining probably the delayed T1-shortening on MRI. The grey matter (especially the cortex) is probably more vulnerable than the white matter, explaining why laminar necrosis most often involves the cortex and sometimes also the deep grey matter. The most frequently reported risk factors associated with laminar necrosis are ischemia/hypoxia, status epilepticus, metabolic changes, and radiation therapy.

## 12. Status Epilepticus

**Figure 25:**
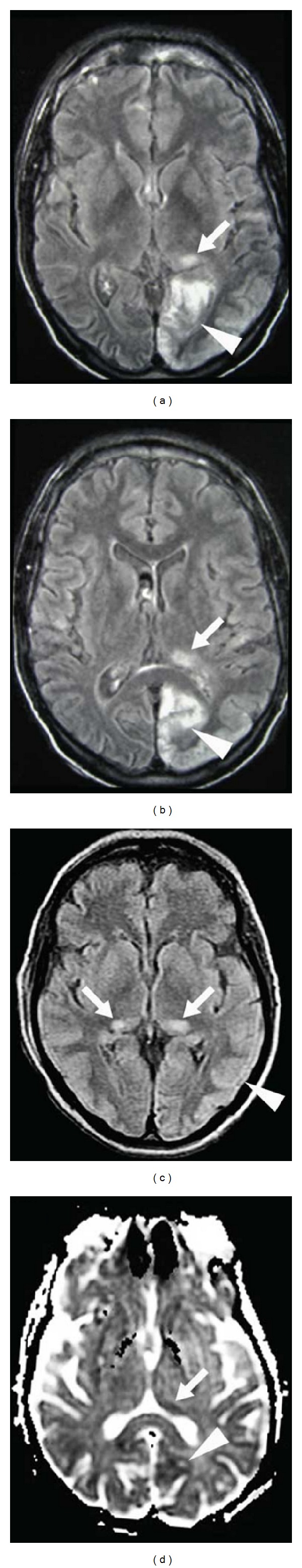
Peri-ictal thalamic lesions (arrows) following status epilepticus in patient 1 (a and b) with partial occipital status epilepticus related to MELAS in the left occipital lobe (arrowheads on a and b) all seen as hyperintensity on FLAIR imaging and in patient 2 (c and d) with generalized status epilepticus with also left occipitotemporal cortical signal changes due to the seizures seen as hyperintensity on FLAIR imaging (c) and as hypointensity on ADC map (d).

Thalamic DWI hyperintense lesions appearing in the region of the pulvinar, ipsilateral to the epileptiform activity, can be seen after prolonged partial status epilepticus (Figure 25). These peri-ictal diffusion abnormalities of the thalamus, likely the result of excessive activity in the thalamic nuclei having reciprocal connections with the involved cortex, are associated with seizure origin in the posterior quadrant and with the presence of ipsilateral cortical laminar involvement on DWI [23].
